# Solution NMR study of the titin I-band IgI domain I82 shows unusual conformational dynamics

**DOI:** 10.1007/s10858-026-00493-2

**Published:** 2026-06-01

**Authors:** Colleen M. Kelly, Shayma Abukar, Matthew J. Gage, Mark Pfuhl

**Affiliations:** 1 Chemistry Department, University of Massachusetts Lowell, Lowell, MA 01854 USA; 2 UMass Movement Center, University of Massachusetts Lowell, Lowell, MA 01854 USA; 3 School of Cardiovascular and Metabolic Medicine and Sciences & Randall Centre, King’s College London, London, SE1 1UL UK

**Keywords:** Protein structure, Tyrosine corner, Immunoglobulin fold

## Abstract

β-sandwich domains of the intermediate immunoglobulin fold (IgI) are key building blocks of many large, multidomain proteins – such as titin. The uniformity of the basic fold of these domains does not seem to be an impediment to their adaptation to a wide variety of functions. IgI domains in different regions of titin have to fulfil distinct functions which is seen in subtle differences of sequence, structure and stability. This is particularly true for the N2A region whose IgI domains are subtly distinct from other parts of I-band titin. We have already shown the unusual structure and properties of IgI domain I83 which is able to bind to calcium and is part of a binding site for F-actin and p94. To continue our exploration of the titin IgI domains of the N2A region we describe here the solution NMR structure of domain I82 of murine titin. The structure of the murine domain is virtually identical to the human homologue. However, the NMR investigation reveals the existence of distinct conformers around a highly conserved glycine, part of the tyrosine corner motif at the junction of the EF-loop with the F-strand. ^15^N relaxation data show substantial line broadening for residues around this glycine, confirming conformational exchange on the fast-intermediate time scale. The unusual dynamics could for the first time explain the high level of conservation of the tyrosine corner glycine via a function in the folding of the domain.

**Supplementary Information:**

The online version contains supplementary material available at 10.1007/s10858-026-00493-2.

## Introduction

Giant multidomain proteins such as titin (Herzog 2018), myosin binding protein C (MyBP-C) (Pfuhl and Gautel 2012), myomesin (Auerbach et al. 1999) or obscurin (Young et al. 2001) fulfil a broad range of functions in striated muscle ranging from the organisation of sarcomeric structures (Linke and Hamdani 2014) to the regulation of muscle contraction (Pfuhl and Gautel 2012) and protein homeostasis (Lange et al. 2005). Despite such a divergent range of functions these proteins appear to fulfil most of them using only a very narrow set of building blocks, namely small (~ 100 amino acids) β-sandwich domains of the fibronectin type III (fnIII) (Main et al. 1992, Campbell and Spitzfaden 1994) or intracellular immunoglobulin (IgI) fold (Holden et al. 1992, Harpaz and Chothia 1994). Since the first structures of the latter were determined (Holden et al. 1992, Pfuhl and Pastore 1995), the fundamental pattern of essential, conserved amino acids for this fold has been well defined (Harpaz and Chothia 1994). The need for a relatively low level of sequence identity around 30% has allowed this fold to adapt to a range of functions involving the interaction with IgI domains of other scaffolding proteins (Pernigo et al. 2010), acting as a ruler (Bennett et al. 2020) and providing flexibility, extensibility and force (Linke et al. 1999) to the interaction with the thin filament (Dutta et al. 2018) and with metals (Nishikawa et al. 2019). The improvement of experimental and theoretical methods has allowed to explore location and interactions of these domains in their native sarcomeric context (Tamborrini et al. 2023) and to predict their 3D structures with high levels of confidence (Jumper and Hassabis 2023). However, some segments of these giant proteins are difficult to observe using microscopy and non-conserved features of structure and dynamics often confound prediction tools.

These considerations are of importance in the study of the N2A region of titin which was shown to have complex functional and structural features such as the interaction with several regulatory proteins, binding to the thin filament and interactions with metal ions (Nishikawa et al. 2019). We were able to demonstrate recently the identification of calcium specific metal binding sites in domain I83 of murine titin (Kelly et al. 2021) while a crystal structure of the I81-I82-I83 tandem of human titin was published shortly afterwards (Stronczek et al. 2021). These complementary experimental methods provide improved insights into the relationship of detailed domain structure and dynamics to the function of the N2A segment. In this context we have performed an NMR study of domain I82 on its own to explore its structure and dynamics independent of its neighbouring domains to develop an understanding of the effects that the tandem arrangement can have on the individual domain structure and vice versa. While our NMR work provides useful information into the relationship between sequential domains it also gives an unexpected insight into the conformational plasticity of the IgI fold through the combined analysis of ^15^N relaxation data and conformers. We find that a short segment of the EF-loop around a highly conserved glycine in the tyrosine corner exists in two conformations that exchange on the fast-intermediate time scale.

## Results

### NMR characterization of I82

Like the neighbouring titin domain I83 (Kelly et al. 2021) also domain I82 gives high quality NMR spectra, allowing the collection of 2D and 3D heteronuclear NMR spectra to obtain a full NMR assignment which covers all amino acids with only the amide resonance of G52 and a small number of side chain resonances missing. A total of 1178 chemical shifts were assigned, corresponding to 84% of the theoretical total. Of all atoms in the domain, 95.7% ^1^H, 99.1% ^15^N and 76.1% ^13^C resonances could be assigned. Based on the combination of Cβ and Cγ chemical shifts (Schubert et al. 2002), all prolines (P4, P9, P35, P50) are in the trans configuration (see Table 1). Their Cβ carbon chemical shifts suggest that all cysteines (C26, C30, C80, C102) are reduced.

**Table 1:** Chemical shift based assignment of trans/cis configuration of proline residues. Trans proline residues have a chemical shift difference of 4.51+- 1.37 while cis-proline has a difference of 9.64 +- 1.62

Residue	Cβ / ppm	Cγ / ppm	Δ(Cβ – Cγ) / ppm
P4	31.8	26.5	5.3
P9	31.9	27.4	4.5
P35	32.1	26.8	5.3
P50	31.2	27.8	3.4

### Solution structure of I82

In the early phases of the structure calculation, it was noticed that a small portion (residues 73–77) of the polypeptide chain did not converge towards a single structure but rather diverged into two distinct conformers. Consequently, several tens of NOEs were removed by Aria due to mutual incompatibility. From there, we identified two distinct sets of NOEs that were compatible with one but not the other conformer and vice versa. This suggested that there are indeed at least two genuine conformers for this portion of the protein. To simplify completion of the structure, explicit sets of distance constraints were generated from those discarded NOEs and separated from the bulk of NOEs compatible with both conformers. Similarly, DANGLE (Cheung et al. 2010) calculation of dihedrals from backbone chemical shifts for this portion of the protein gave odd results (see Table 4), difficult to reconcile with the NOE derived conformation. Therefore, for residues 73–77 no backbone dihedral constraints were used. Structure calculation was completed separately for the two conformers using the structure restraints compatible with both conformers plus one set each of conformer specific distance and H-bond constraints. One conformer has this part of the chain bulging outwards so that it is designated as I82OUT, the other has this part of the chain shifted towards the interior of the protein and so is designated I82IN. Outside this small region the two conformers are essentially identical so that the global description of the structure will be given using the I82IN conformer as representative with only the differences being addressed specifically for each of them.

**Fig. 1:**
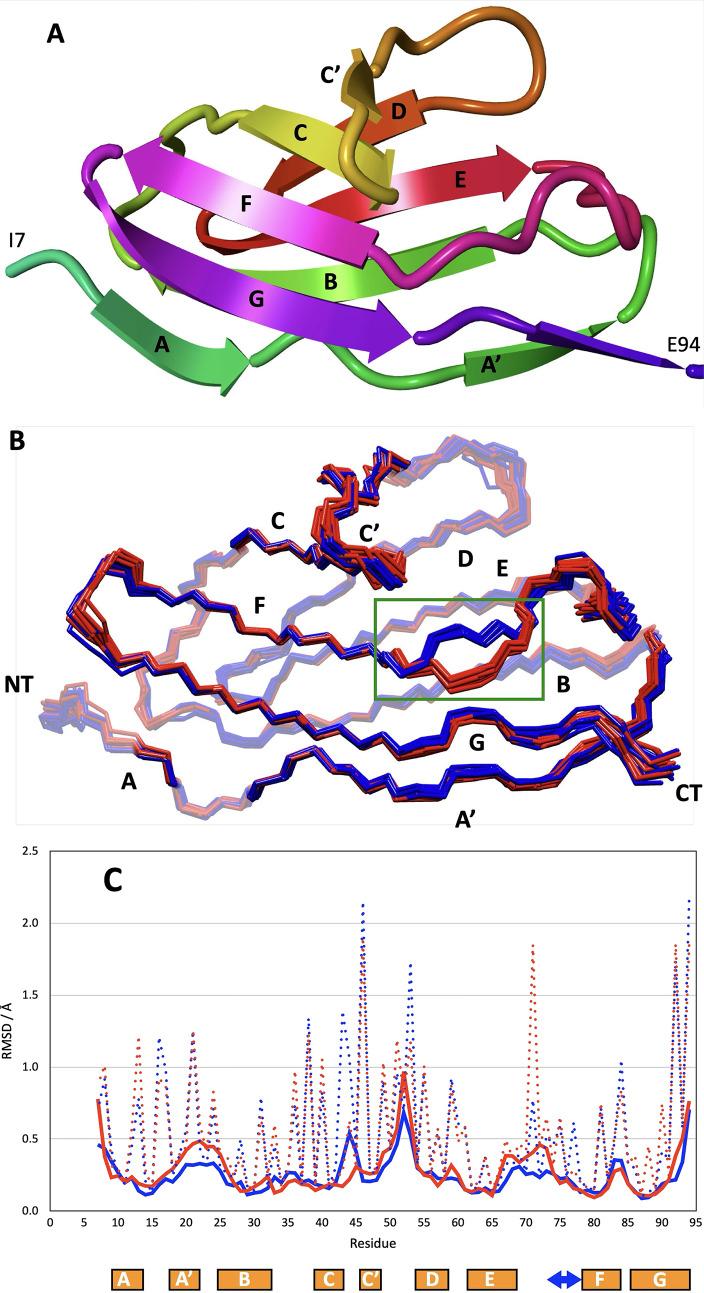
**A)** Best representative structure (I82IN) shown in cartoon mode, coloured by residue number (N- to C-terminus: green to violet) with the disordered segments G1-T6 and G96-C102 removed. **B)** Superposition of the 10 best backbone (N, Ca, C’) structures each for I82IN (red) and I82OUT (blue). The area of the conformational heterogeneity is marked by a green rectangle. **C)** RMSD plot for the two conformers, I82ΙΝ is coloured red, I82OUT is coloured blue. Backbone (N, Ca, C’) RMSD values are shown with solid lines, all heavy atom RMSD values are shown in dotted lines. Only residues of the native I82 (I7-E94) in the construct are shown. Secondary structure elements are shown below the plot including the tyrosine corner as blue arrow

The overall structure of I82IN is shown in Fig. 1A showing the typical IgI domain fold. The family of structures for both conformers is shown in Fig. 1B. The backbone RMSD for both conformers is around 0.3 Å and all heavy atom RMSD around 0.8 Å (Fig. 1C) indicating the high quality of the data used (see Table 2 for structural statistics for both conformers). As expected, lowest RMSD values are seen for regions with regular secondary structure while higher backbone RMSDs are seen at the termini while even the most disordered loop (CD-loop) has a backbone RMSD of only 0.6 Å (Fig. 1C). Quality control using ProCheck (Laskowski et al. 1996), WhatCheck (Hooft et al. 1996) and Molprobity (Williams et al. 2018) do not reveal any significant problems apart from the mostly disordered E94 and K95 which show distorted bond angles (See Fig. S1-S4). In both conformations > 95% of the residues are in the most favoured and allowed regions. Experimental restraints are well reflected in the family of structures with hardly any distance violation larger than 0.3 Å or dihedral violation larger than 5^o^ (Table 2). The structure (see Fig. 1A) has the expected features for an IgI domain with the highly conserved β-sandwich fold comprised of two sheets with four strands each, the typical β-bulge in strand A and the strand switch-over from one to the other sheet between strands A and A’ which makes a parallel sheet with strand G.

The structures of both conformers have been submitted to the PDB: I82IN: 9IBI, I82OUT: 9IBK.

**Table 2:** Structural statistics for both conformers of I82. Violation values are averages over the family of 20 final structures

Parameter	I82 IN	I82 OUT
Distance constraints	1812	1805
Dihedral constraints	154	154
H-bond constraints	50	47
Distance violations > 0.3 Å	1.7 +- 1.0	1.6 +- 1.1
Distance violations > 0.1 Å	55.2 +- 5.2	54.6 +- 4.3
RMS of distance violations / Å	0.034	0.033
H-bond violations > 0.1 Å	0	0
RMS of H-bond violations / Å	0.014	0.019
Dihedral violations > 5^o^	1.2 +- 0.4	0.9 +- 0.5
RMS of dihedral violations / ^o^	4.37 +- 1.06	3.38 +- 1.81
Ramachandran most favoured %	70.1	72.9
Ramachandran allowed %	25.9	24.4
Heavy atom RMSD (6–96) / Å	0.72	0.77

### Conformers in the EF-loop

**Fig. 2:**
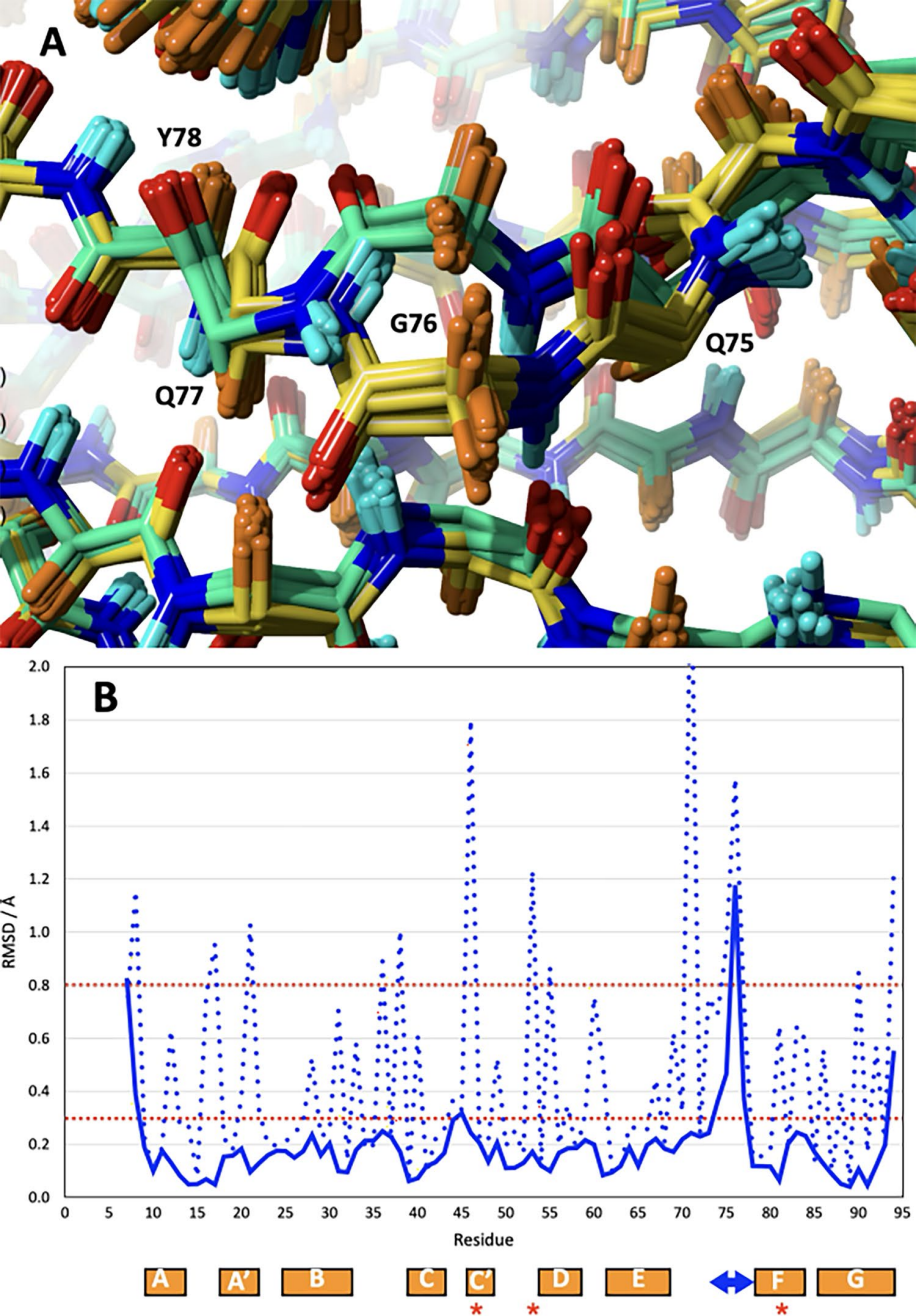

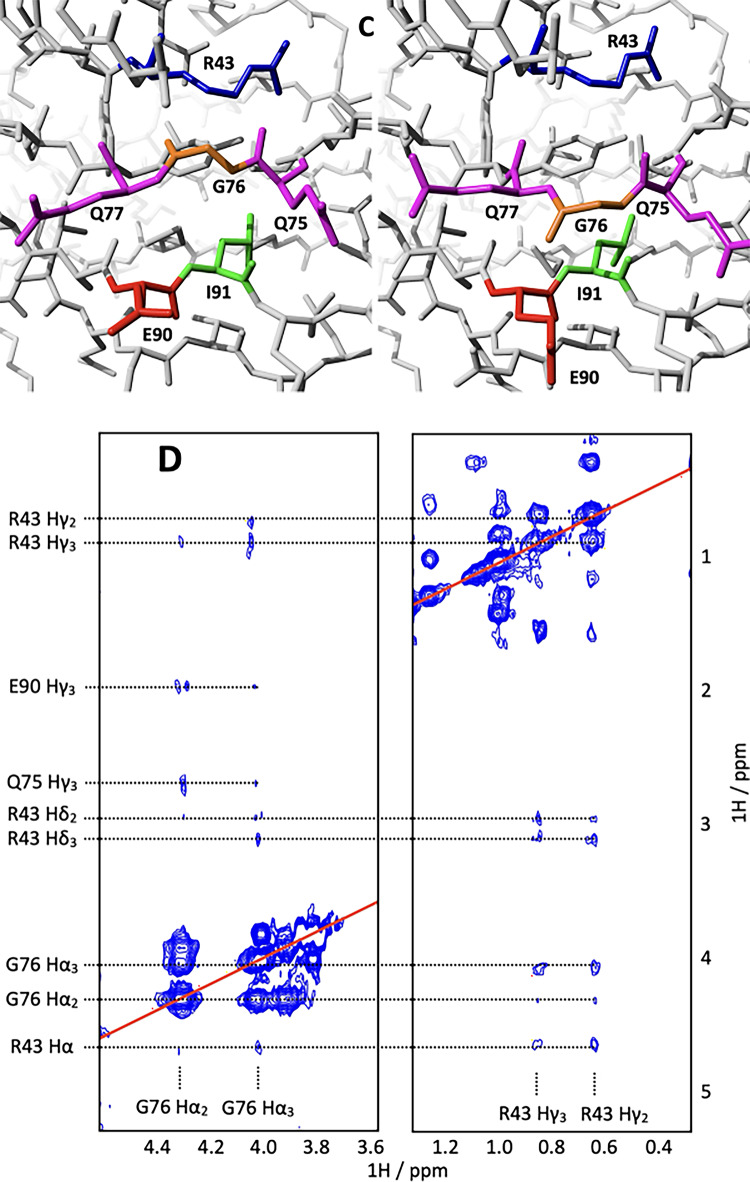
Exploration the two conformers at the junction of EF-loop and F-strand. **A)** Detailed view of the backbone of the two different conformers for the superposed structures within the green rectangle from Fig. 1B. Atoms are coloured according to element: Oxygen: red; nitrogen: blue, α-hydrogens: orange, carbons in I82IN: light green, carbons in I82OUT: light yellow. **B)** Plot of the residue specific RMSD values (backbone N, Ca, C’ solid lines, heavy atoms dotted lines) of the comparison of the best I82IN with the best I82OUT conformer. **C)** All atom structures are shown separately for I82IN (left) and I82OUT (right). Selected amino acid side chains are coloured by property (apolar: green; polar: magenta; negatively charged: red; positively charged: blue; glycine: orange) and labelled. **D)** Portions of the 3D ^13^C resolved NOESY experiment showing NOEs indicative of the two conformers. Left: ^1^H-^1^H plane taken at the Cα chemical shift of G76. Right: ^1^H-^1^H plane taken at the Cγ chemical shift of R43

The two conformers identified during the NMR structure calculation have their main differences in the EF-loop around residues 73–77 (Fig. 2A and B). The two conformers result from sets of NOEs that are compatible with one of these conformers but not with the other one. The biggest difference is seen for G76 which has at the same time NOEs from its α-protons to backbone and sidechain protons of e.g. R43, E90 and Q75 which are mutually incompatible (Fig. 2C and D). The relevant protons of these two groups of amino acids are on average more than 10 Å apart while the distances from G76 were calibrated in the range of 2–4 Å. This would have made it quite difficult to satisfy all of these distances simultaneously in the same structure. Similarly, Q77 has HN NOEs to the β-protons of R43 but also strong cross peaks to Q90 (see Table 3).

Overall, the two conformers have a backbone RMSD of only 0.24 Å and an all-heavy atom value of 0.65 Å. The detailed backbone and all-heavy atom RMSD plot in Fig. 2C confirms virtually identical structures apart from the immediate vicinity of G76 plus a small backbone variation in the neighbouring CD-loop where G45 has a backbone RMSD of just over 0.3 Å.

**Table 3:** Conformation specific NOE derived distances for the region of the EF-loop showing conformational heterogeneity (residues A73-Q77). Distance restraints for each conformer are shown together with the actual distances in the best experimental structures

Conf.	Atom 1	Atom 2	Restraint	IN	OUT	Difference
OUT	HB1 D74	HH Y78	2.0	2.9	2.3	0.6
	HB1 Q75	HA A72	2.5	3.7	2.7	1.0
	HA2 G76	HG1 E90	3.0	5.1	3.3	1.8
	HA2 G76	HG2 E90	4.5	6.2	4.6	1.6
	HN G76	HA R92	3.5	5.2	3.6	1.6
	HD2 R43	HA D74	2.0	3.2	2.1	1.1
IN	HB3 D74	HG2# I67	3.5	3.6	4.1	0.5
	HA Q75	HG L91	2.5	2.7	3.5	0.8
	HG1 R43	HA3 G76	2.5	2.6	5.8	3.2
	HG2 R43	HA3 G76	2.0	2.3	5.3	3.0
	HD2 R43	HA3 G76	3.0	3.3	6.1	2.8
	HD1 R43	HA3 G76	4.0	4.3	7.1	2.8
	HA R43	HA2 G76	4.0	4.1	6.6	2.5
	HD21 N44	HA2 G76	2.0	2.3	3.6	1.3
	HD21 N44	HA3 G76	3.5	3.7	5.1	1.4
	HN N44	HA2 G76	4.0	4.1	7.0	2.9
	HN N44	HA3 G76	3.5	3.6	5.6	2.0
	HE21 Q77	HA A89	3.5	3.6	7.6	4.0
	HA3 G76	HE# Y78	4.0	4.3	7.3	3.0
	HB3 D74	HG1# V70	4.0	4.1	6.3	2.2

Significant sidechain variations elsewhere are seen for R46, R53 and R71 all of which are in immediate spatial vicinity of the region of conformational heterogeneity. There is some variation at the termini, but this is part of the overall variability of the two structural families (see Fig. 1B). Therefore, only variations well above this threshold can be considered important. The largest difference between the conformers is seen for the peptide group between G76 and Q77 (Fig. 2A ) which rotates by almost 180^o^ between the two forms (changes in the ψ-angle of G76 and the φ-angle of Q77 by ~ 150^o^) resulting in a shift of the position of its Cα by 2.5 Å. Consequently, the carbonyl oxygen of G76 points inwards in I82IN and outwards in I82OUT. While it is exposed to solvent in I82OUT it can form a hydrogen bond with the backbone amide proton of N44 in I82IN. Conversely, the amide proton of Q77 is pointing inward in I82OUT but outward in I82IN. In both of these orientations it is able to form H-bonds with the solvent. In I82OUT this is facilitated by the outward bulging of the polypeptide chain creating space for solvent to enter. The peptide group between Q75 and G76 is shifted a bit but retains its orientation. In I82OUT the amide proton of G76 is able to form an H-bond with L91 while in I82IN it is pointing into solution to form H-bonds with the solvent. The sidechains of Q75 and Q77 are somewhat shifted but retain their general position, pointing away from the domain, towards the solvent (Fig. 2C). In contrast, the G76 Cα group makes close hydrophobic contacts with the sidechain of R43 in the I82IN conformation. Conversely, the Cα group of G76 is fully exposed in the I82OUT conformation.

### Protein dynamics

**Fig. 3:**
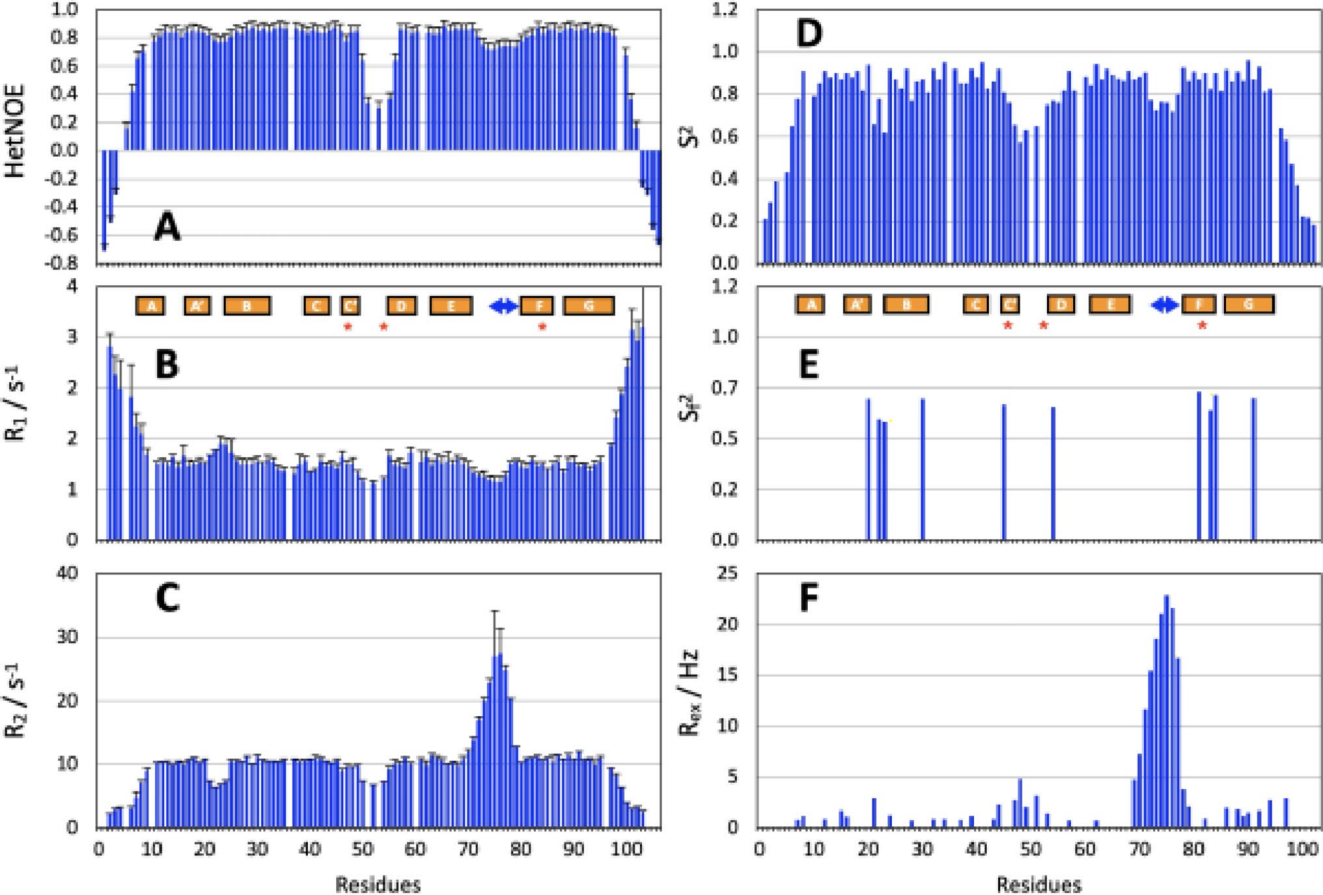
^15^N relaxation analysis of domain I82. All values are shown as a function of residue position. Secondary structure elements (boxes), the area of the conformational heterogeneity (arrow) and the amino acids different between human and murine I82 (stars) are indicated. **(A)** ^1^-^15^ ^1^H-^15^N NOE. **(B)** ^15^N R_2_. **(C)** ^15^N R_1_. **(D)** S^2^. **(E)** S_f_^2^ **(F)** R_ex_

The dynamics of I82 was characterised by ^15^N relaxation measurements (^15^N R_1_, R_2_ and ^1^H-^15^N heteronuclear NOE shown in Fig. 3A-C). Analysis of the ^15^N R_1_/R_2_ ratios allows the determination of the overall rotational correlation time τ_c_ = 6.6 +- 0.3 ns, close to the expected vale of 5.9 ns based on the molecular weight. As often happens with this type of domain (Kelly et al. 2021, Pfuhl et al. 1997, Ratti et al. 2011) despite a reasonable axially symmetric shape (ratio of principal axes 1.00:0.98:0.55), using an axially symmetric diffusion model did not significantly improve the fitting of the experimental data (p > > 0.05), so that a model for spherical symmetry was used. Observation of the raw relaxation data shows that I82 is mostly a rigid, compact domain with only the extended N- and C-termini showing very high levels of mobility on the ps time scale with negative NOEs, high R_1_ and low R_2_ values.

Within the domain only the CD loop shows higher levels of mobility on the ps time scale. Most interesting is part of the EF-loop which shows modestly lower NOEs and R_1_ values but significantly increased R_2_ values for residues 73–77. The detailed analysis of local dynamics using the Lipari-Szabo approach (Fig. 3D-F, full table of results and motional models Fig. S9) confirms the high level of local dynamics at the termini and – to a lesser extent - in the BC and CD loops. Most interestingly, the complex dynamics in the EF-loop can be dissected into a minor component of fast ps-level motion as indicated by the slightly lowered order parameter S^2^. Additionally, the large values of the exchange contribution R_ex_ clearly indicate the presence of intermediate time scale dynamics in the region around residues 73–77. To ensure that the exchange line broadening was not caused by self-association (Pfuhl et al. 1999) the experiments were repeated at ~ 1/3 of the original concentration and showed no significant differences (data not shown).

### Comparison of NMR and X-ray structure

**Fig. 4:**
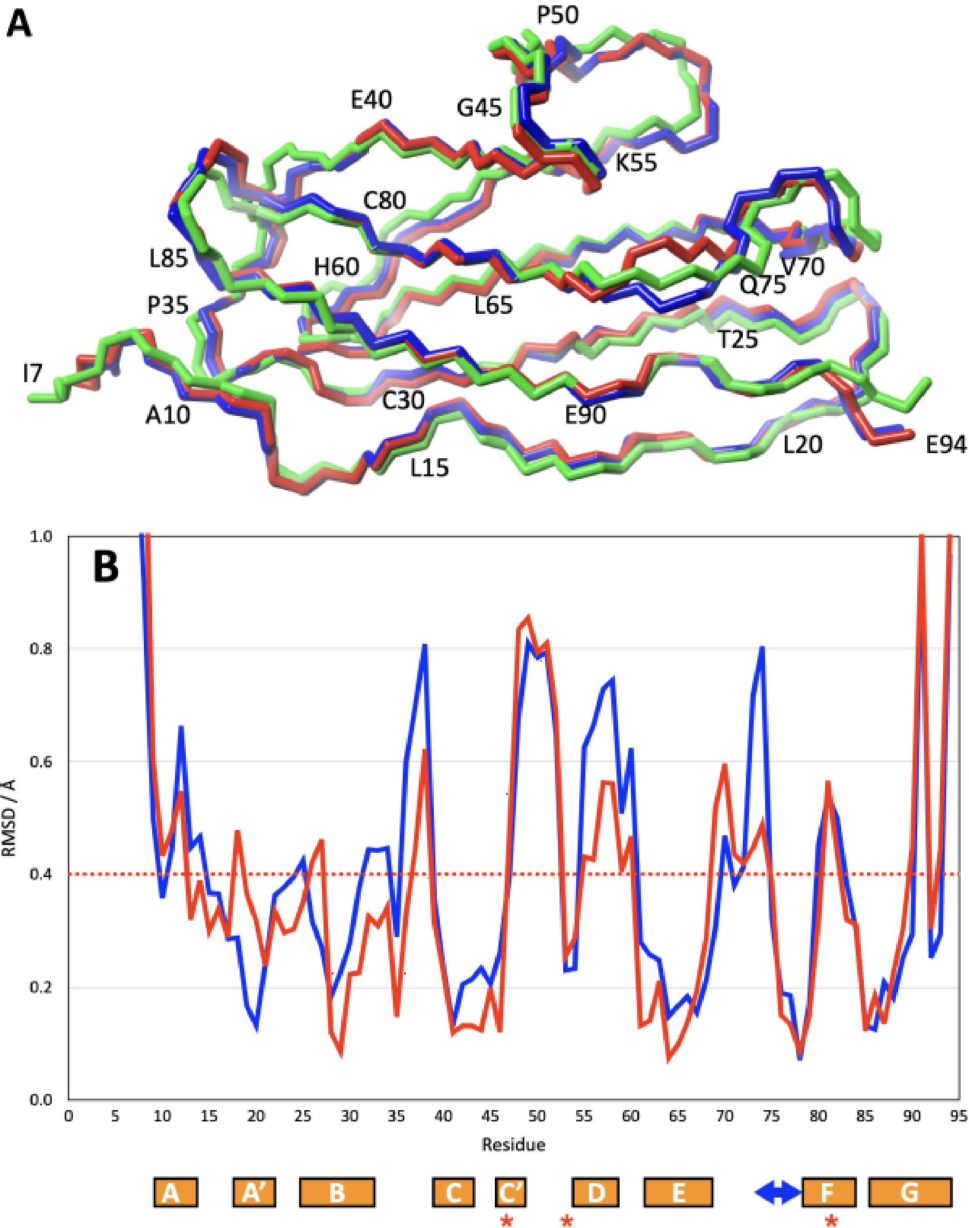
Comparison of the solution structure of mouse I82 with the crystal structure of human I82 in the context of the tandem construct I81-I82-I83. **(A)** superposition of I82 in crystal (green) on the NMR structure (I82IN, red and I82OUT, blue). **(B)** Backbone RMSD plot for the structure superposition of the NMR structure of I82IN (red) and I82OUT (blue) onto the crystal structure of I82. The magnitude of the uncertainty in both X-ray and NMR structures is indicated by the red dotted line

Recently, the crystal structure of the *H. sapiens* titin tandem I81-I82-I83 was determined by crystallography (Stronczek et al. 2021). This offers the interesting opportunity to compare the structure of domain I82 in the context of its nearest neighbours in the sequence to its structure in isolation. Apart from three positions, the sequences of mouse and human I82 are identical. In position 47 the mouse version has a Val, the human version an Ile, in 54 it is Val vs. His and in 82 it is His vs. Tyr. The NMR structure of mouse I82 is very similar to the crystal structure of its human equivalent in the tandem construct (backbone RMSD to copy A in 7AHS I82IN = 0.47 Å, I82OUT = 0.48 Å, see Fig. 4A). This compares very well to the ~ 0.4 Å RMSD between the four copies of I82 in the crystal and the RMSD of the NMR structure determination (~ 0.3 Å). In other words, the overall structural differences of human and mouse I82, alone in solution and in the context of I81/I83 in the crystal are on the same magnitude as the experimental uncertainties of the respective methods. A detailed view of the structural differences is seen in the backbone RMSD plot in Fig. 4B. The only noticeable but rather modest differences are seen at the N- and C-terminus and in the CD, EF and FG loops.

### Conservation of the EF-loop

**Fig. 5:**
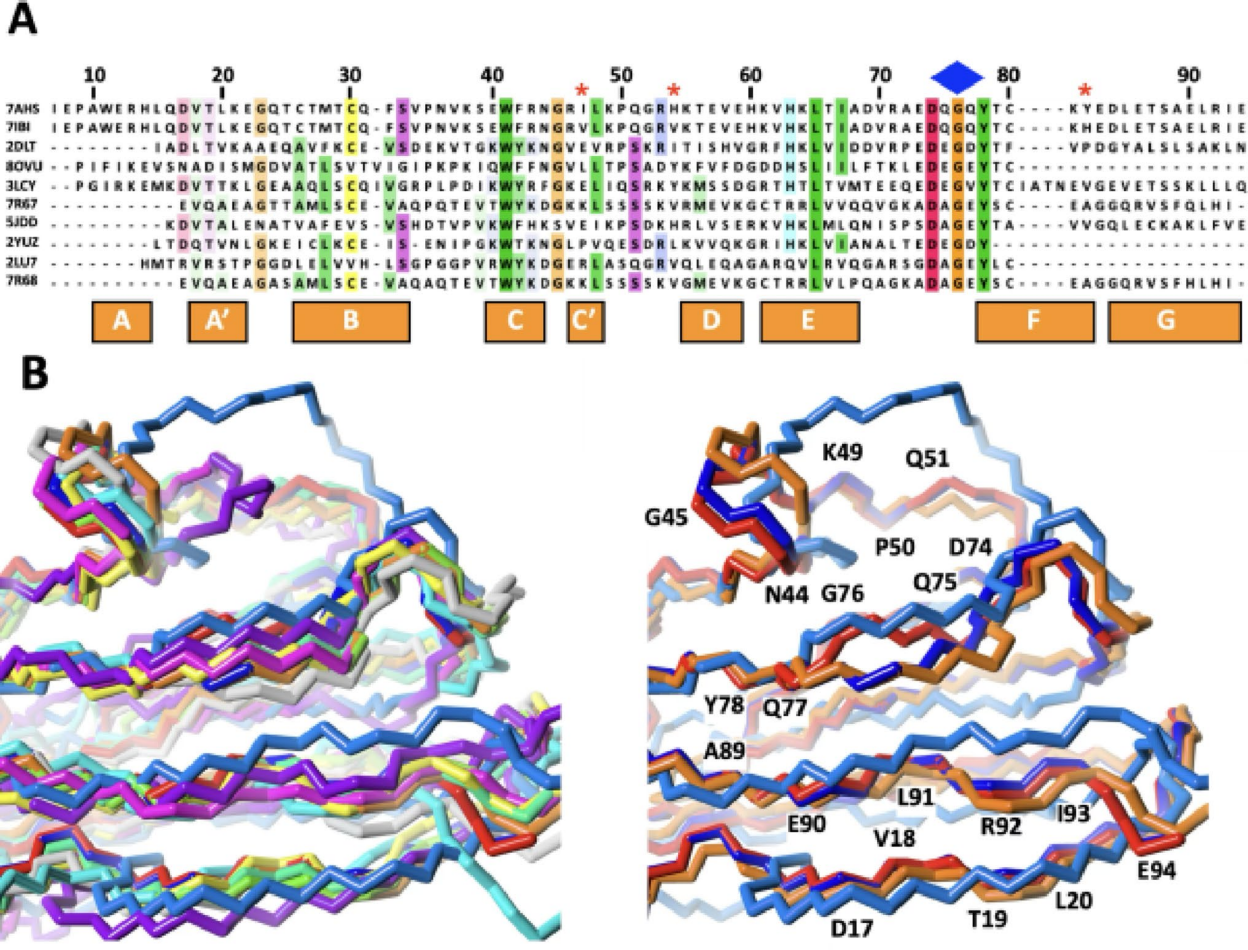
**(A)** Sequence alignment of IgI domains similar to murine I82 with known high resolution experimental structures. Amino acids are coloured by property (as in Fig. 2D) and level of conservation (30% identity threshold). Sequences are identified by the corresponding PDB code: **7AHS**: human titin, domain I82. **9IBI**: mouse titin, domain I82. **2DLT**: mouse myosin binding protein-C (MyBP-C), fast isoform, domain C3. **8OVU**: human titin, domain I21. **3LCY**: human titin, domain A164. **7R67**: human obscurin, domain Ig13. **5JDD**: human titin, domain I9. **2YUZ**: human MyBP-C, slow isoform, domain C4. **2LU7**: human obscurin like 1, domain Ig11. **7R68**: human obscurin, domain Ig12. **(B)** Structure superposition of the domains aligned in A). 2DLT: yellow; 2LU7: cyan; 2YUZ: magenta; 2YCU: grey; 5JDD: orange; 7AHS: turquoise; 7R67: light blue; 7R68: dark violet; 8OVU: green; I82_IN_: red; I82_OUT_: dark blue. Left: overview of all domains. Right: only I82IN, I82OUT, 5JDD and 7R67. Selected residues of I82 are labelled

The sequence of I82 was compared to similar IgI domains with known structure identified from a sequence similarity search in the PDB. The 8 additional domains show a remarkable level of sequence conservation at the end of the EF-loop and the transition towards the F-strand. D74, G76 and Y78 (which form the tyrosine corner typical for the IgI fold) are perfectly conserved in all these domains (Fig. 5A) having previously been identified as key residues of the I-set of Ig domains (Pfuhl and Pastore 1995, Harpaz and Chothia 1994). It is therefore unlikely that the conformational heterogeneity identified in mouse I82 is caused by an unusual local sequence variation. Comparing the structures of these domains (Fig. 5B) shows a surprising structural heterogeneity for this short portion of sequence, despite its high level of sequence conservation. From this small sample we can identify structures that almost perfectly match the two conformers of I82 described here: 7R67 (obscurin Ig13) matches I82IN and 5JDD (titin I9) matches I82OUT. The conservation of the glycine in a key position of this region might predispose this short sequence to adopt various conformations depending on contacts with the rest of the protein and thus depending on variations elsewhere in the sequence such as in the CD-loop which is nearby.

## Discussion

The solution structure of domain I82 from murine titin provides an interesting glimpse into conformational plasticity of proteins that is rarely seen, mostly due to technical limitations. Structures from X-ray crystallography provide a snapshot of one conformation that is compatible with the constraints imposed by crystal contacts while electron microscopy is as yet limited with regards to smaller proteins and conformational heterogeneity. NMR in contrast is well suited to the observation of conformational heterogeneity as the protein under study is not externally constrained to a single conformation. It is also possible to infer conformational dynamics indirectly from the measurement of ^15^N relaxation. However, there is a need for different conformations to be defined by sufficiently distinct conformational constraints so that differences can be seen. The latter is even more important when the conformational exchange is on the fast-intermediate time scale. For exchange on this timescale the lifetime of a particular conformation can be estimated to be between 100 us − 10 ms (Palmer 2004). Such a lifetime is well above the minimum of 1 ns needed for the buildup of an observable NOE (Neuhaus and Williamson 1989) as documented extensively in the case of water-protein NOEs (Otting and Wuethrich 1989, Otting et al. 1991). Observation of distinct conformations is therefore not limited to slow exchange. The only difference is that for fast-intermediate exchange, the NOEs will be the average of all conformations sampled. As the key NOEs of interest here can only be observed in one of the two resolved conformations the net effect of conformational averaging is a reduction in peak intensity by about ½ (assuming roughly equal populations of the two conformers).

Even though only a small number of amino acids is involved in the conformational heterogeneity of I82, the differences between the two conformations observed are just about large enough to create sets of NOE distance constraints that are mutually incompatible (Fig. 2D; Table 3). In the context of the conformational dynamics, the low intensity of these peaks can be easily explained by severe line broadening as well as reduction in intensity as described above.

**Table 4:** Overview of backbone torsion angles in the two conformations of the EF-loop. Shown are the φ/ψ angles of the two conformers of I82 (IN/OUT), the dihedrals predicted from the chemical shifts using DANGLE (Pre), selected backbone chemical shifts in ppm and the total number of NOEs for each residue, including both general and conformer specific (the latter are listed explicitly in Table 3)

Res	IN		OUT		Pre		Chemical shifts / ppm				NOE
	φ	ψ	φ	ψ	φ	ψ	*N*	Cα	Cβ	Hα	
E73	-83	-18	-81	-21	-63	-44	114.67	58.44	28.62	4.13	15
A74	-78	4	-99	20	-70	-35	117.31	55.38	42.50	4.51	21
Q75	-84	-178	-110	166	-85	-7	119.66	57.30	29.97	4.05	31
G76	121	84	132	-70	94	-4	110.00	44.62	-	4.03/4.32	22
Q77	-9	109	-163	137	-69	138	120.06	55.02	29.91	4.75	23
Y78	-83	138	-118	139	-119	126	130.96	57.45	41.42	5.48	33

Conformational averaging under fast-intermediate dynamics affects different NMR parameters in different ways, and it is essentially only NOEs that can clearly point towards distinct conformations provided that they are sufficiently divergent. As a matter of fact, it cannot be excluded that the EF-linker region might contain more than the two IN and OUT conformers. It is simply that other conformers didn’t leave a clear enough mark on the pattern of NOEs. All other conformation specific NMR parameters will appear as their population weighted averages which significantly reduces their usefulness to meaningfully contribute to resolve two or more conformers. It can be seen clearly in Table 4 that the averaged chemical shifts and thus the backbone dihedral angles derived from them via DANGLE do not correspond to any of the conformations compatible with the NOEs. Similar averaging would be expected for the measurements of scalar or dipolar couplings. As a result, measurement of coupling constants would not be helpful to further resolve the conformers observed through NOEs. As was shown (Clore and Schwieters 2006), these average couplings would need to be reconstructed from a population weighted ensemble of conformers which makes it difficult to accurately resolve the number and shape of those conformers.

The NMR structure of the isolated murine I82 is remarkably similar to the crystal structure of the human version as part of the I81-I82-I83 tandem. The overall variability of around 0.4 Å is comparable to the uncertainty in either experimental structure determination and is therefore insignificant. Local variations larger than the experimental uncertainty are limited to the termini and some loops. And even in the latter they are at most 0.8 Å in the CD and EF-loops. It is actually intriguing that there is not more of a difference in the loops involved in sequential domain-domain contacts of I82 with adjacent I81/I83 in the crystal structure. However, a close analysis of the sequential domain-domain contacts (see supplementary material, Fig. S5) shows that these are rather small with only 163.5 Å^2^ of solvent exposed surface covered between I81-I82 and 198.8 Å^2^ covered between I82 and I83. Such modest interfaces are on the small side compared to e.g. side by side contacts between IgI domains such as seen between titin M10 and Obscurin Ig1^12^ which is much larger (593.6 Å^2^). It is therefore unlikely that these limited interdomain contacts of I82 in the crystal structure would have much of an effect of the conformation of I82.

The conformers in the EF-loop that we observe in murine titin domain I82 by NMR in solution are not seen in any of the four copies of the domain in the crystal structure of human I82 (Fig. 3, Fig. S6). While average B-factors are quite different for the four copies of I82 in the crystal they are not particularly higher in the EF-loop in any of them. Also, there are only three residues different between murine and human domain I82 (Fig. 5A) and all of them are far from the EF-loop and G76. It is therefore unlikely that the conformational heterogeneity observed by NMR is caused by sequence differences between murine and human titin I82. Instead, it is more probable that crystal contacts could play a role in influencing the conformation around G76. Part of the EF-loop and the F, G, and C-strands plus part of the CD-loop form part of a major crystal contact between two copies of the titin molecule (Fig. S7 & S8) covering a substantial solvent accessible surface of 556 Å^2^ between I82 and I81.

Despite the high level of sequence conservation (Harpaz and Chothia 1994)(Fig. 5A) this part of the protein shows a broad range of shapes ranging from conformations very similar to the I82IN all the way to I82OUT. As a matter of fact, in this small sample of similar domains, it was possible to find IgI structures with a conformation of the EF-loop virtually identical to I82IN (7R67) as well as to I82OUT (5JDD). The IgI fold requires only a low level of sequence identity so that the selected domains represent a broad range of sequences, each of which could indirectly affect the EF-loop via direct contacts with residues in this loop as well as by modestly affecting the 3D arrangement of the key secondary structure elements. It is therefore clear that despite virtually identical sequences the EF-loop of the IgI fold can arrange itself in a broad range of conformers defined by the overall sequence context of the entire domain.

Residue G76 is highly conserved across the intermediate set of immunoglobulin domains together with the tyrosine corner (Hemmingsen et al. 1994) and is part of the original definition of this fold (Harpaz and Chothia 1994) as position F1. However, the authors of this article were unable to link this very high level of conservation to any crucial role played by this glycine in the structure of the domain. This is in stark contrast to e.g. G23 in I82 (position A’B in the I-set (Harpaz and Chothia 1994)) which is equally well conserved and necessary to allow a tight turn at the end of strand A’ by assuming a ‘forbidden’ combination of φ/ψ angles (see Fig. 5A). It might therefore be possible that the conservation of G76 has another reason which could be related to protein folding. Folding of β-sandwich and Greek Key domains has been studied extensively (Fowler and Clarke 2001). It was found that strands B, E & D combine early into the initial nucleus of tertiary interactions (Hamill et al. 2000). The other strands including strand F follow later (Geierhaas et al. 2006). A flexible hinge between the E and F strands might be required to allow the early native-like structures to assemble into the fully native structure. This could be provided by G76 and the mobility we see in the folded domain may be a remnant of such a function. The folding kinetics of the closely related domain I27 was studied by mutagenesis of many residues (Wright et al. 2003) but unfortunately not including the equivalent of G76. It therefore remains to be seen how important the conservation of G76 in the I-set F1 position is for protein folding.

## Methods

### Molecular biology & protein expression

The expression construct for mouse titin IgI domain Ig82, comprising residues 8811–8898 (according to GenBank entry NM_0011652.3 for mouse titin; the equivalent residues in the inferred complete (IC) sequence of human titin are 9987–10071). Mouse I82 was cloned into vector pET151/D-TOPO using Invitrogen’s TOPO cloning system. Proteins for NMR analysis were expressed and purified in a manner identical to that described recently for domain I83 (Kelly et al. 2021). The cloning procedure has added the sequence GIDPFT at the N-terminus and the sequence KGELRSGC at the C-terminus. To simplify residue numbering, we count the number of residues from the first residue in the construct - G1 - so that the first genuine amino acid of I82 – I8811 – is I7 in the construct and the last original residue is E94. To simplify the comparison with the crystal structure of the equivalent domain I82 from human titin (Stronczek et al. 2021) the same construct numbering was applied to this one as well. The first residue of human I82, I9987, will therefore also be numbered as I7 to match the numbering of the mouse construct used in this work.

### NMR spectroscopy

NMR spectra were collected essentially as described recently (Kelly et al. 2021). In short, spectra (^15^N HSQC, and ^15^N NOESY-HSQC 100 ms (800), ^15^N TOCSY-HSQC 60 ms (700), HNCA (700), HNCACB (800), HN(CO)CACB (700), HN(COCC)H (800), HN(COC)C(H) (800), aromatic and aliphatic ^13^C-HSQC (950) & ^13^C NOESY-HSQC 100 ms (950); using standard pulse sequences provided by the manufacturer) were collected at T=298 K using Bruker Neo spectrometers (equipped with TCI cryoprobes) at fields indicated after each experiment using samples of ~ 0.5mM concentration in a buffer made of 20 mM sodium phosphate, 50 mM sodium chloride, 2 mM dithiothreitol and 0.02% sodium azide, pH 7.0 with a volume of ~ 300 µL in a Shigemi tube with 5% D_2_O for the lock. Data were processed in Topspin 4.0 and analysed using CCPNMR 2.4.2 and 3.2.12 (Skinner et al. 2016; Mureddu et al. 2020). The resulting assignment has been deposited in the BMRB, accession code 34980 & 34981.

^15^N relaxation was studied at a magnetic field strength of 800 MHz using ^15^N T_1_, ^15^N T_2_ and ^1^H-^15^N heteronuclear NOE spectra. 10 data points were recorded for the T1 measurement: 50, 100, 200, 300, 400, 500, 600, 800, 1200, 1500 ms using the Bruker pulse sequence hsqct1etf3gpsi3d. This experiment utilises double refocused INEPT transfers (Bax et al. 1969) coupled to an inversion recovery block for ^15^N relaxation during which protons are decoupled using a modified DIPSI sequence (Rucker and Shaka 1989). Phase sensitivity in F1 and water suppression is achieved using echo/anti-echo-TPPI gradient selection (Tyburn et al. 1992). The T2 experiment was recorded with 10 data points: 16.96, 33.92, 50.88, 67.84, 84.8, 101.76, 118.72, 135.68, 152.64, 169.6 ms using the Bruker pulse sequence hsqct2etf3gpsi3d. This experiment utilises double refocused INEPT transfers coupled to a CPMG block (Kay et al. 1989) for ^15^N relaxation incorporating ^1^H 180^o^ pulses for ^1^H decoupling. Phase sensitivity in F1 and water suppression is achieved using echo/anti-echo-TPPI gradient selection. For the ease of processing and analysis the T_1_ and T_2_ experiments are recorded in a pseudo-3D manner. Peak volumes were automatically calculated and then fitted to a single exponential decay curve in CCPNMR. Errors for the T_1_ and T_2_ values are the errors of the exponential fit. Heteronuclear NOEs were measured with the Bruker pulse sequence hsqcnoef3gpsi. NOE values calculated as the ratio of cross peak volumes in the spectrum with ^1^H saturation (3 s) over the spectrum without ^1^H saturation. Phase sensitivity in F1 and water suppression is achieved using echo/anti-echo-TPPI gradient selection. The error for the heteronuclear NOE was taken as the difference in the values from repeating the experiment once. Experimental errors in R_1_, R_2_ and NOE were directly propagated in the calculation of the rotational diffusion coefficient and indirectly into the LSA analysis via the calculation of critical χ_2_ values. As a consequence, the selection of more elaborate models will need not only to satisfy an F-statistic (models 1,2,3) but also produce a χ2 value well below the critical one which is calculated from the experimental errors in R_1_, R_2_ and heteronuclear NOE. This ensures appropriate appreciation of the errors in the experimental data. The structure of I82 has principal axes of 1.00:0.98:0.55 (calculated with PDBinertia (Mandel et al. 1995, Palmer et al. 1991) using only the rigid residues I7-G96). However, fitting of the R_2_/R_1_ ratios with a spherical and axial symmetric model using R2R1diffusion (Mandel et al. 1995, Palmer et al. 1991) did not result in a significant differences of the χ2 values by the latter (χ2 sphere = 19.2, χ2 axial = 18.6, F = 1.21, p > > 0.05). Therefore, the rotational correlation time τ_c_ - describing the timescale of rotational diffusion for the protein as a whole - was calculated from a fit of R_1_/R_2_ ratios for all residues with heteronuclear NOEs > 0.6 using a spherical model of motion. Subsequently, model free analysis according to Lipari & Szabo (Lipari and Szabo 1982, Lipari and Szabo 1982, Clore et al. 1990) was performed using home written scripts in R (described previously (Pfuhl et al. 1997)). Essentially, the combination of global τ_c_ and individual R_1_, R_2_ and NOE values are fitted for each individual amino acid using a range of motional models. In the simplest model (model 1) only one parameter, the order parameter S^2^, is used. It ranges from values of 0 to 1, describing the amplitude of motion on the ps time scale without explicit assumption about a precise time constant. A value of 0 means complete freedom of movement, while a value of 1 means complete absence of any movement. For amino acids, where this simple model does not give satisfactory results, more complex descriptions of local motion can be used by the introduction of further parameters. In cases where R_1_ and NOE can be fitted well using only S^2^ but where R_2_ is comparatively high one can introduce a correction factor called R_ex_ that compensates under the assumption that exchange line broadening due to fast-intermediate dynamics leads to the increase of R_2_ values (model 2). In cases where the ps timescale motions are somewhat slower one can introduce an explicit time constant τ_e_ that improves fitting of all three experimental parameters (model 3). Where each of these two modifications are insufficient to improve fitting of the data, they can also be combined (model 4). Finally, it is known that local motion on the ns time scale can occur, albeit rarely. Where relaxation parameters cannot be fitted by any of the other models one can assume ns time scale local motions which introduces an additional order parameter S^2^_f_ (range 0–1) and an additional time constant for movement on the ns time scale, τ_i_ (model 5) (Clore et al. 1990). Selection of models 1,2 & 3 uses a rigid F-statistic allowing the selection of a more complex model only if the improvement in the fit is shown to be significant by a p-value < 0.05^26^. Due to the number of parameters involved, selection of models 4 & 5 cannot be based on such statistics. Instead, the critical χ_2_ value is calculated for each model using the experimental errors. If models 1–3 are not satisfactory, model 4 or 5 can only be selected if their χ_2_ is lower than the corresponding critical χ_2_. Usually, these criteria a so stringent that for most residues models 1–3 give a satisfactory representation of the experimental data while models 4 or 5 are rarely used.

### Structure calculation & analysis

Structure calculation was performed using the AriaWeb server (Allain et al. 2020, Brünger et al. 1998) via a NEF file (Gutmanas et al. 2015) exported from CCPNMR assign 3.2.12. Distance restraints were obtained from cross peak intensities in ^15^N and ^13^C resolved NOESY spectra (100ms mixing time, ^13^C aliphatic and aromatic spectra were recorded separately) and calibrated automatically in Aria. Tolerances for matching chemical shifts in peak assignments were 0.03 ppm for ^1^H and 0.3 for ^15^N and ^13^C and the peak intensity was used for calibration. Distance constraints discarded by Aria for violations were analysed manually and, where suitable, included as additional, conformation specific distances for either of the two conformers (IN/OUT). For these restraints original calibrations from Aria were used with only upper distance limits, adjusted for attenuation by line broadening and averaging, allowing for a maximal error of 0.3 Å in either of the two conformations. Dihedral constraints were obtained from Dangle (Cheung et al. 2010) using Cα, N, Cβ, Hα chemical shifts and were used with variable allowances depending on the structure. For residues in regular secondary structures an allowed range of ± 20.0^o^, for irregular but rigid regions ± 30.0^o^ was used. No dihedral restraints were used for residues 73–77. It was aimed at having a maximum of 5.0^o^ violation of dihedral constraints. Hydrogen bond restraints were introduced once basic convergence was attained where H-bonds were observed in 15 out of the best 20 structures in the family of structures. Structure calculation was performed using default parameters apart from calculating 50 structures at each step and then proceeding with the best 10 of them into the next iteration. At the final iteration the best 20 structures were selected and refined in water. The selection criteria were a combination of violation of experimental constraints and structure quality which is the Aria default. All other Aria parameters were the default settings of the server. Once divergence was noticed in the region surrounding G76, distance restraints for the relevant region 73–77 were split in two batches according to compatibility with one or the other conformation. They were added to subsequent calculations of one or the other structure as explicit unambiguous restraints maintaining the original calibration by ARIA.

All structures were analysed, superimposed and displayed in Yasara Structure 24.4.10 (Krieger and Vriend 2014).

### Sequence analysis

The native sequence of murine titin domain I82 was used to search the PDB using the Blastp server (Altschul et al. 1997) for IgI domains with known structure and similar sequence. 8 of the 10 top hits of the search were used to produce an alignment in Jalview 2.11 (Waterhouse et al. 2009) using MuscleWS (Edgar 2004) with default settings. Entry 8G4L was removed because it is a cryoEM structure of the thick filament and thus does not have the resolution required to capture the details of the EF-loop. Entry 8P35 was left out because it is a mutation of the wild type entry 8OVU and thus essentially a duplication.

## Supplementary Material

Below is the link to the electronic supplementary material.

Supplementary Material 1

## Data Availability

Data generated in this study consists of NMR spectra assignments as well as the structures of the two conformers of titin domain I82. The assignment has been submitted to the BMRB where it is accessible from entries 34980 and 34981. The structures have been submitted to the PDB where they are accessible from entries 9IBI and 9IBK.
