# Supplementary material for: Solution NMR study of the titin I-band IgI domain I82 shows unusual conformational dynamics
Source: J Biomol NMR. 2026 Jun 1;80(1):14. doi: 10.1007/s10858-026-00493-2 (PMC13226425; doi:10.1007/s10858-026-00493-2)
Supplement: Supplementary file 1 — Supplementary Material 1 [file 10858_2026_493_MOESM1_ESM.pdf]

Fig. S1

WhatCheck Analysis of I82IN

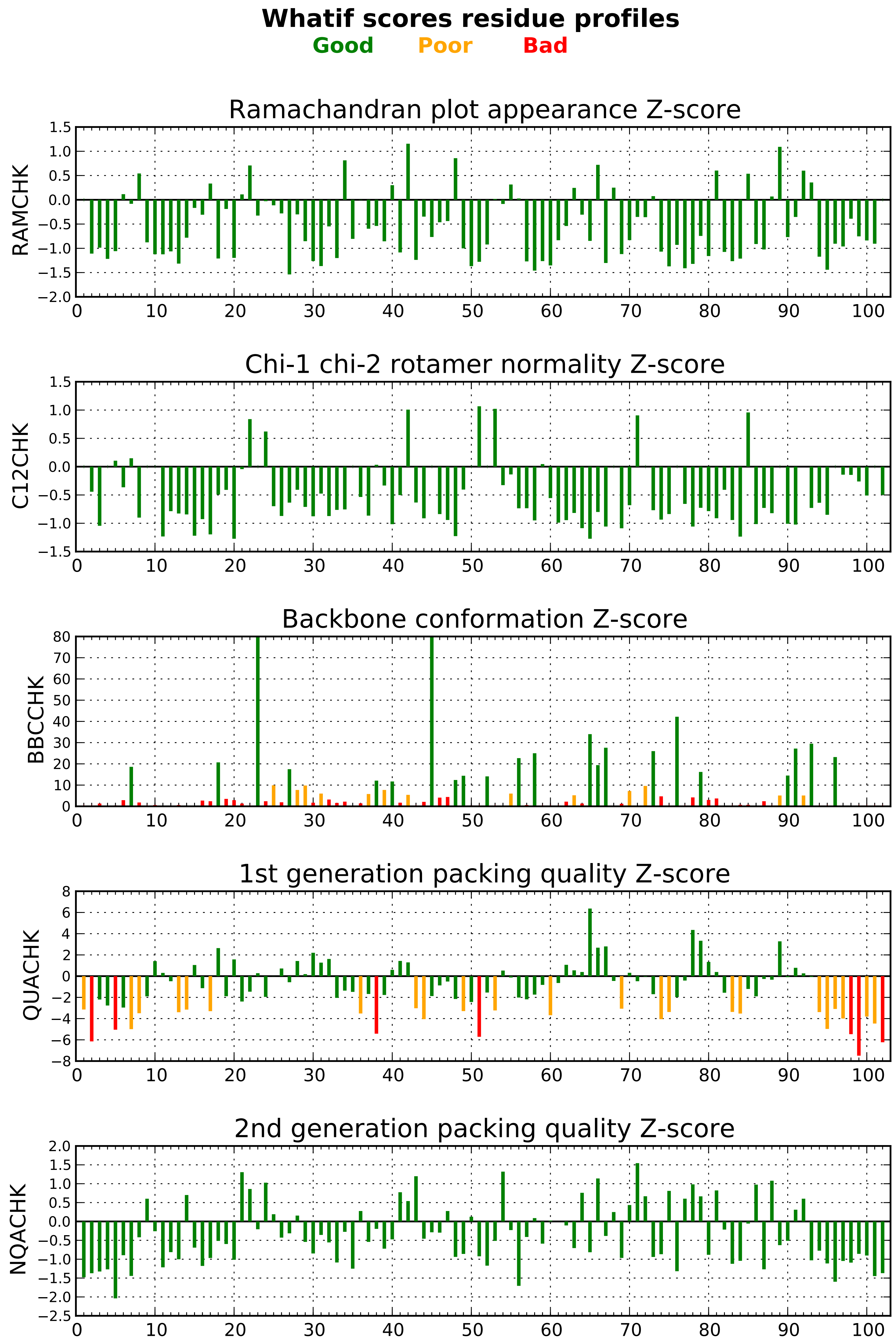

Fig. S2

WhatCheck Analysis of I82OUT

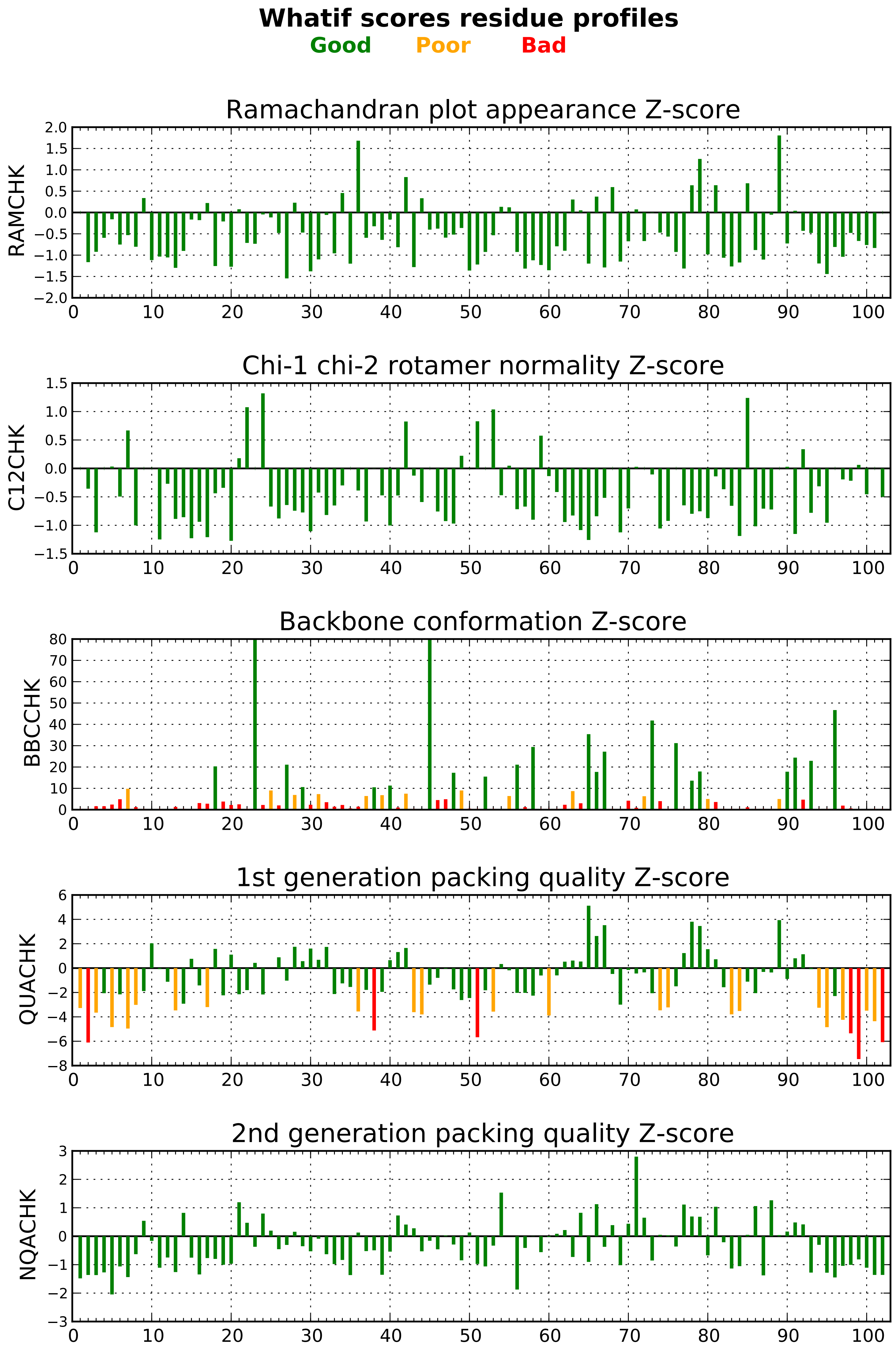

Fig. S3

MolProbity Ramachandran Analysis of I82IN

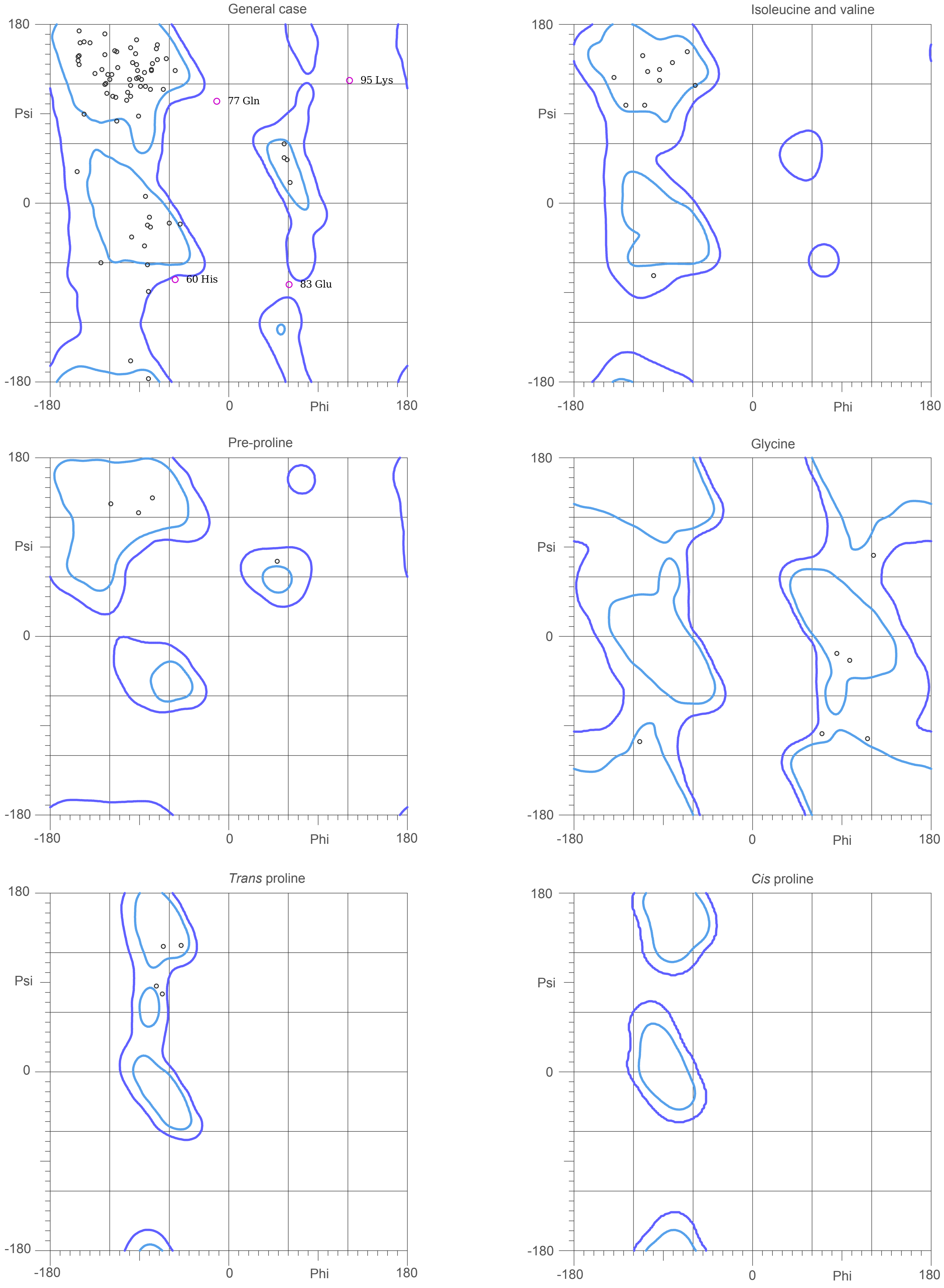

Fig. S4

MolProbity Ramachandran Analysis of I82OUT

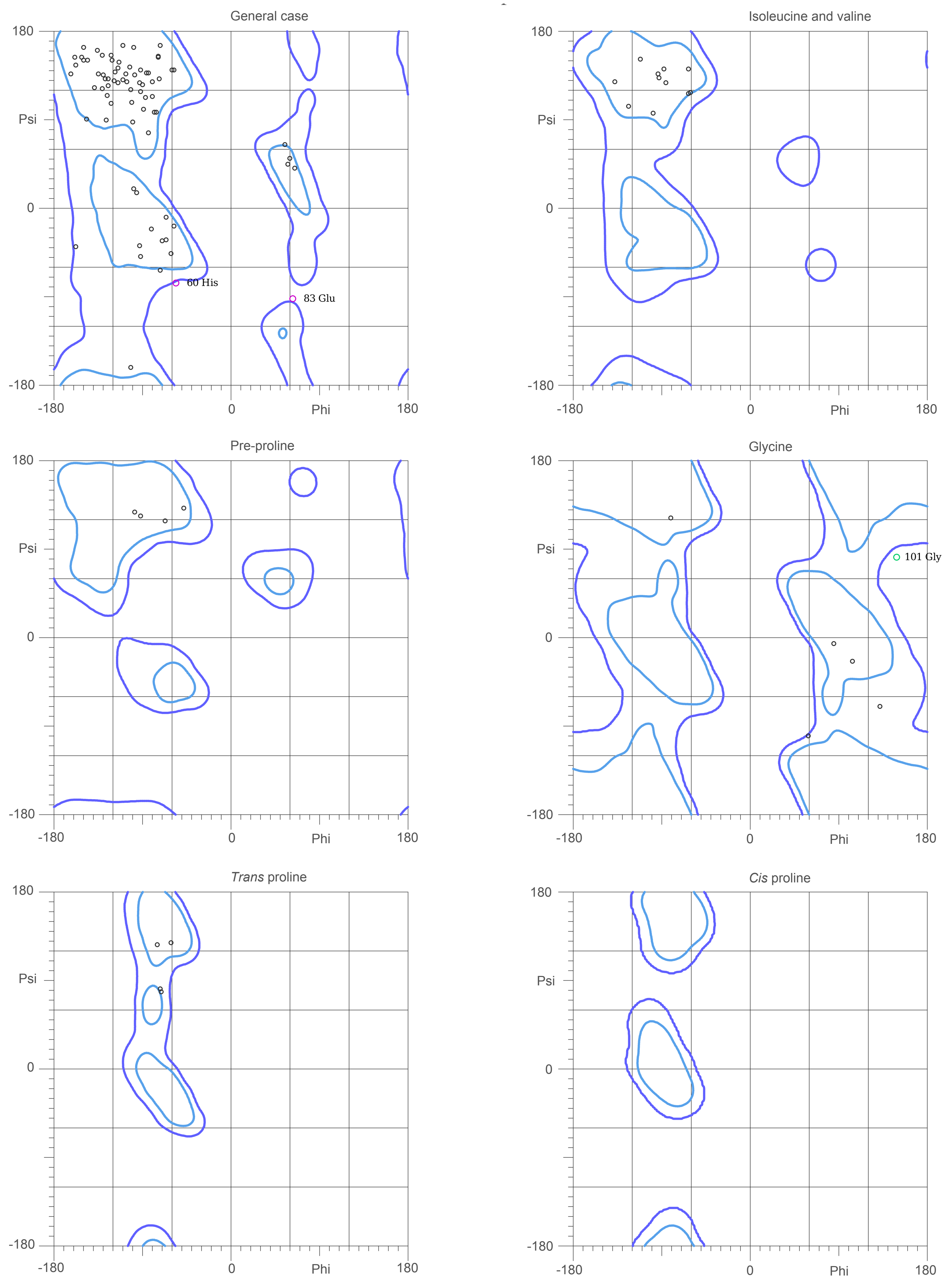

Fig. S5

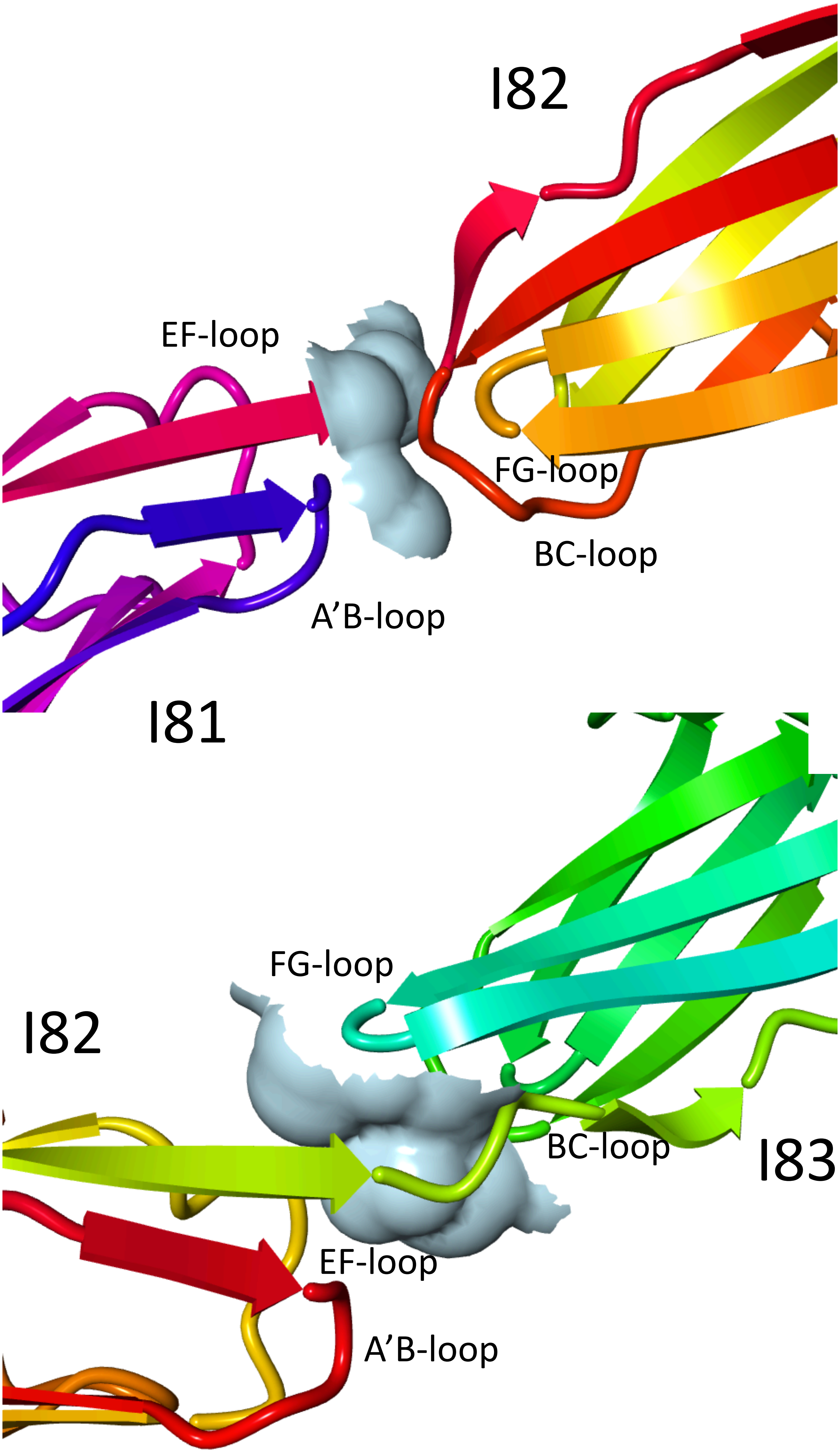

Solvent accessible surface covered in the tandem interfaces of the crystal structure of human titin I81-I82-I83. Top: I81-I82; bottom: I82-I83. Important loops are indicated.

Fig. S6

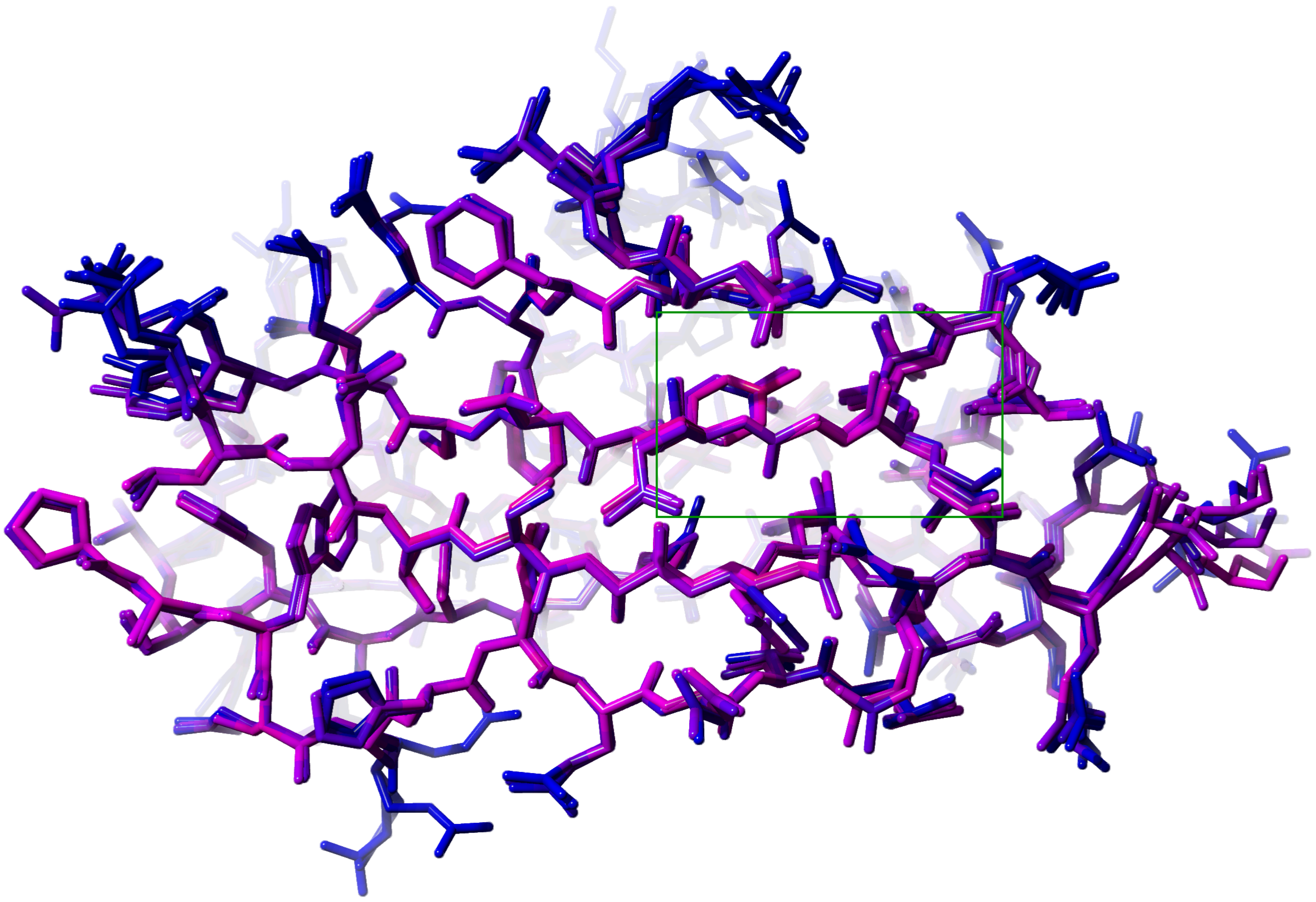

Superposition of the four different copies of I82 in the crystal structure coloured by B-factor (low=red, high=blue). There are no significant local variations in the region of conformational heterogeneity in the NMR study (EF-loop an beginning of F-strand, indicated by green rectangle). The only differences are higher B-values on the periphery and lower ones in the centre as well as different average B-factors in different models.

Fig. S7

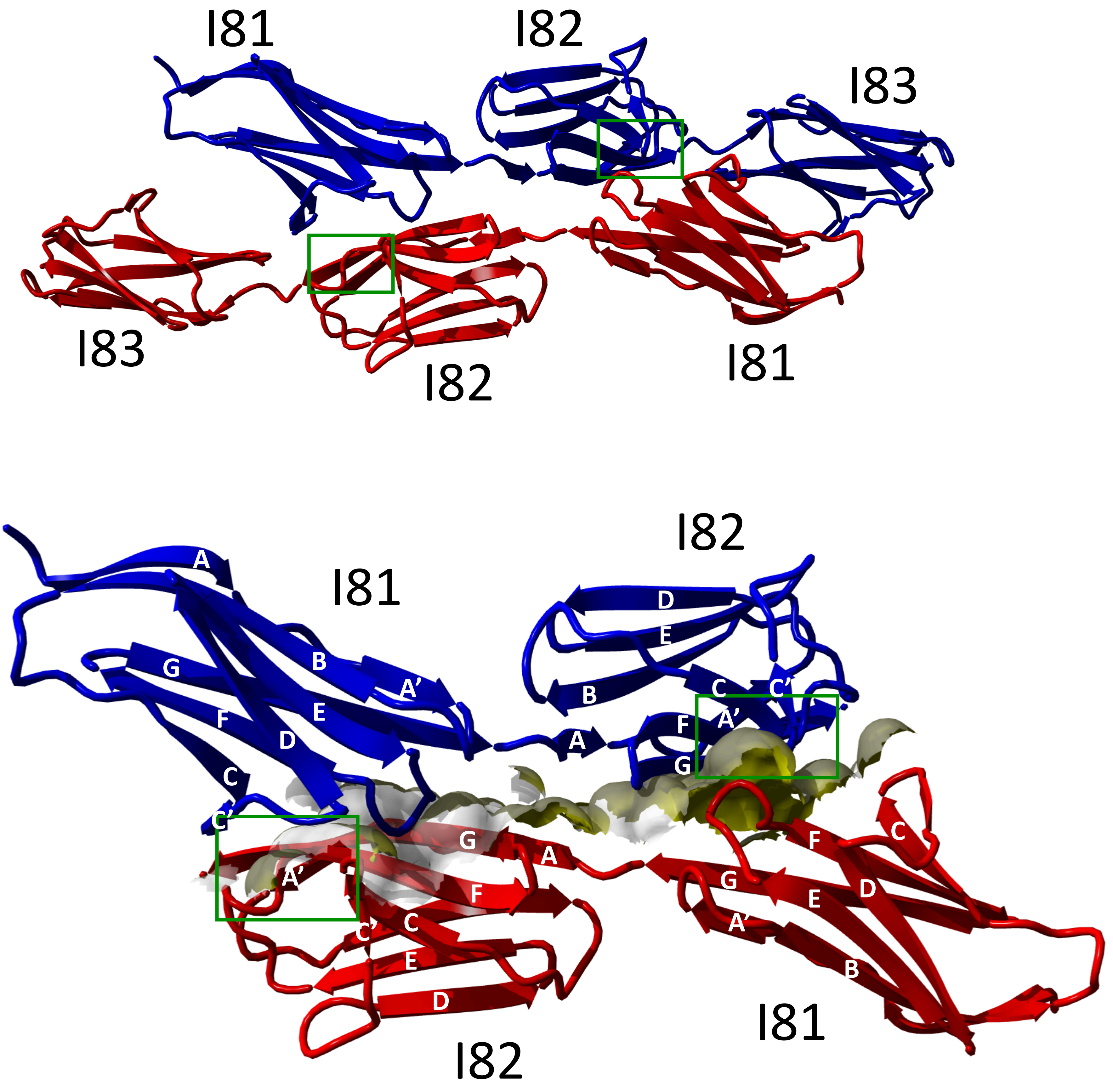

Antiparallel packing of two copies of the I81-I82-I83 fragment in the crystal. The regions around G76 in I82 are indicated by green rectangles and are at the interface between I82 in one molecule and I81 in the other one. Detailed view of the secondary structure elements involved in the contact between I81 and I82 on opposite molecules (I83 removed for simplification). In a fairly symmetric arrangement I81 contacts I82 with the CD and EF-loops plus parts of the F, G and C-strand.

Fig. S8

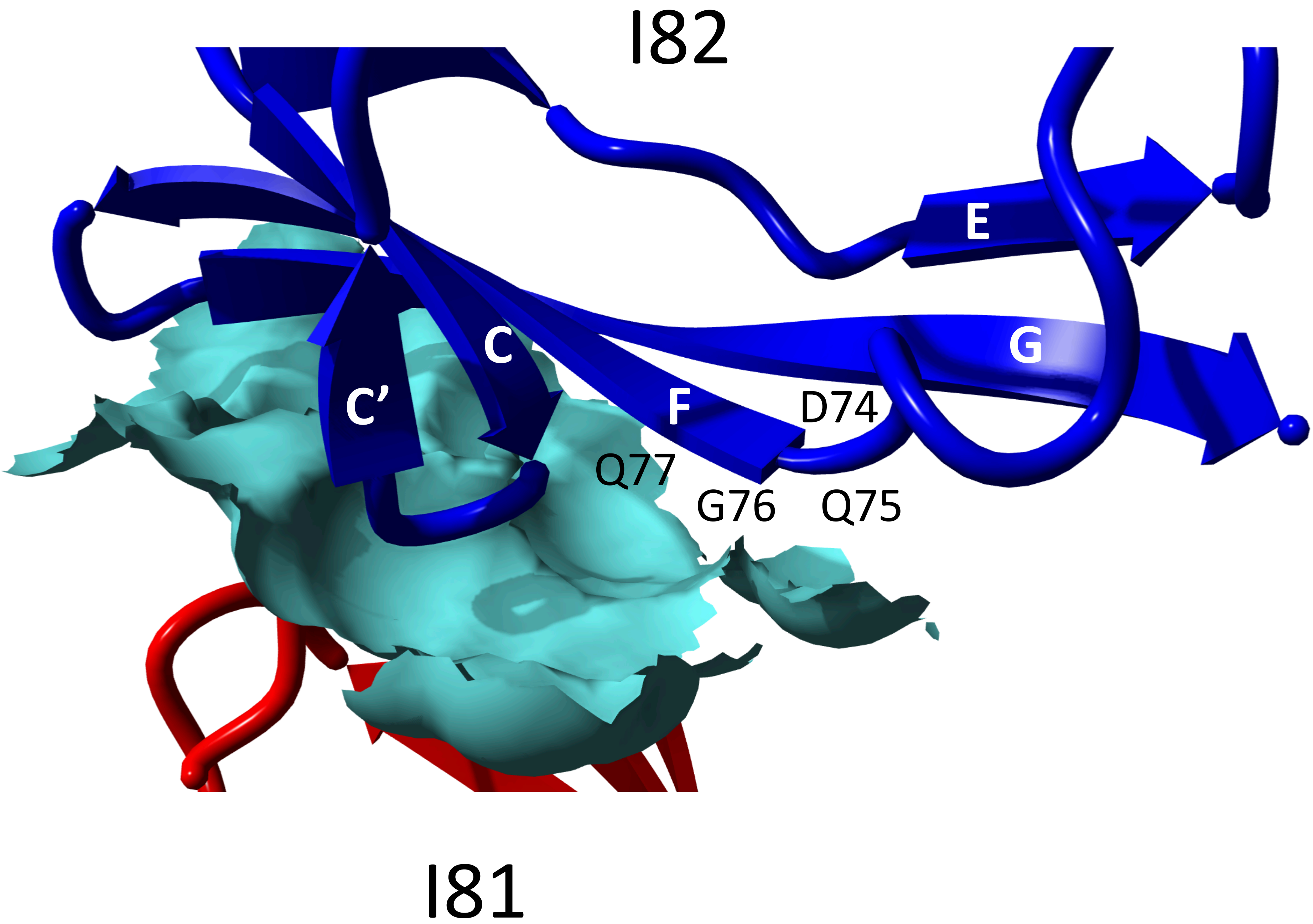

Detailed view of the secondary structure elements involved in the contact between I81 and I82 on opposite molecules (I83 removed for simplification). In a fairly symmetric arrangement I81 contacts I82 with the CD and EF-loops plus parts of the F, G and C-strand.

Table S1

Results of LSA

residue	model	S^2	S_f^2	τ_e / ps	R_{ex} / Hz	τ_i / ns
1	3	0.21	0.00	400	0.00	0.00
2	3	0.29	0.00	410	0.00	0.00
3	3	0.39	0.00	390	0.00	0.00
4						
5	3	0.43	0.00	120	0.00	0.00
6	3	0.65	0.00	180	0.00	0.00
7	4	0.78	0.00	72	0.87	0.00
8	4	0.91	0.00	49	1.24	0.00
9						
10	3	0.79	0.00	29	0.00	0.00
11	3	0.85	0.00	39	0.00	0.00
12	2	0.91	0.00	0	0.83	0.00
13	3	0.88	0.00	22	0.00	0.00
14	1	0.90	0.00	0	0.00	0.00
15	2	0.87	0.00	0	1.67	0.00
16	2	0.90	0.00	0	1.13	0.00
17	3	0.88	0.00	41	0.00	0.00
18	1	0.91	0.00	0	0.00	0.00
19	3	0.82	0.00	21	0.00	0.00
20	5	0.94	0.67	0	0.00	0.73
21	4	0.66	0.00	420	2.90	0.00
22	5	0.78	0.57	0	0.00	1.23
23	5	0.62	0.56	0	0.00	2.42
24	2	0.92	0.00	0	1.25	0.00
25	3	0.87	0.00	0	0.00	0.00
26	1	0.83	0.00	0	0.00	0.00
27	1	0.92	0.00	0	0.00	0.00
28	2	0.77	0.00	0	0.68	0.00
29	1	0.86	0.00	0	0.00	0.00
30	5	0.87	0.67	0	0.00	0.33
31	3	0.81	0.00	22	0.00	0.00
32	2	0.92	0.00	0	0.87	0.00
33	1	0.87	0.00	0	0.00	0.00
34	2	0.95	0.00	0	0.88	0.00
35						
36	1	0.92	0.00	0	0.00	0.00
37	2	0.85	0.00	0	0.80	0.00
38	3	0.85	0.00	20	0.00	0.00
39	4	0.92	0.00	34	1.15	0.00
40	3	0.88	0.00	33	0.00	0.00
41	1	0.95	0.00	0	0.00	0.00
42	1	0.83	0.00	0	0.00	0.00
43	2	0.86	0.00	0	0.84	0.00
44	2	0.92	0.00	0	2.27	0.00
45	5	0.81	0.64	0	0.00	0.25
46	3	0.76	0.00	47	0.00	0.00

Table S1

Results of LSA

residue	model	S^2	S_f^2	τ_e/ps	R_{ex}/Hz	τ_i/ns
47	2	0.66	0.00	0	2.71	0.00
48	4	0.57	0.00	60	4.80	0.00
49	4	0.63	0.00	88	2.05	0.00
50						
51	4	0.65	0.00	192	3.17	0.00
52						
53	4	0.75	0.00	281	1.46	0.00
54	5	0.77	0.63	0	0.00	0.39
55	3	0.76	0.00	88	0.00	0.00
56	1	0.82	0.00	0	0.00	0.00
57	2	0.91	0.00	0	0.74	0.00
58	3	0.82	0.00	43	0.00	0.00
59						
60	1	0.88	0.00	0	0.00	0.00
61	1	0.84	0.00	0	0.00	0.00
62	2	0.94	0.00	0	0.71	0.00
63	3	0.87	0.00	22	0.00	0.00
64	1	0.92	0.00	0	0.00	0.00
65	1	0.89	0.00	0	0.00	0.00
66	1	0.87	0.00	0	0.00	0.00
67	3	0.86	0.00	61	0.00	0.00
68	1	0.91	0.00	0	0.00	0.00
69	2	0.87	0.00	0	4.75	0.00
70	2	0.88	0.00	0	7.26	0.00
71	2	0.90	0.00	0	11.71	0.00
72	2	0.77	0.00	0	15.49	0.00
73	2	0.72	0.00	0	18.62	0.00
74	4	0.76	0.00	398	21.02	0.00
75	2	0.76	0.00	0	22.88	0.00
76	2	0.72	0.00	0	21.63	0.00
77	4	0.80	0.00	0	16.71	0.00
78	4	0.93	0.00	0	3.80	0.00
79	2	0.86	0.00	0	2.08	0.00
80	1	0.91	0.00	0	0.00	0.00
81	5	0.87	0.71	0	0.00	0.55
82	4	0.90	0.00	21	0.88	0.00
83	5	0.82	0.61	0	0.00	0.74
84	5	0.90	0.68	0	0.00	1.28
85	3	0.82	0.00	11	0.00	0.00
86	2	0.92	0.00	0	1.93	0.00
87	1	0.86	0.00	0	0.00	0.00
88	2	0.91	0.00	0	1.82	0.00
89	1	0.86	0.00	0	1.18	0.00
90	4	0.96	0.00	13	1.51	0.00

Table S1

Results of LSA

residue	model	S^2	S_f^2	τ_e/ps	R_{ex}/Hz	τ_i/ns
91	5	0.87	0.67	0	0.00	1.30
92	2	0.93	0.00	0	1.70	0.00
93	3	0.81	0.00	12	0.00	0.00
94	2	0.83	0.00	0	2.75	0.00
95						
96	3	0.64	0.00	153	0.00	0.00
97	4	0.58	0.00	210	2.91	0.00
98	3	0.47	0.00	180	0.00	0.00
99	3	0.37	0.00	260	0.00	0.00
100	3	0.22	0.00	310	0.00	0.00
101	3	0.22	0.00	340	0.00	0.00
102	3	0.18	0.00	412	0.00	0.00

Table with the results of the Lipari-Szabo analysis of ^{15}N relaxation data. The parameters used in the fitting of the relaxation data for individual residues are shown together with the model selected for each amino acid and the error. Residues 4, 9, 35 & 50 are proline so that no values can be reported for this measurement. For residues 52, 59 & 95 no relaxation data could be analysed mainly because of poor S/N. The models use the following parameters: model 1: S2; model 2: S2 & Rex; model 3: S2 & tau_e; model 4: S2, Rex, tau_e; model 5: S2, Sf2, tau_i.
